# Characterization of the mechanism of drug-drug interactions from PubMed using MeSH terms

**DOI:** 10.1371/journal.pone.0173548

**Published:** 2017-04-19

**Authors:** Yin Lu, Bryan Figler, Hong Huang, Yi-Cheng Tu, Ju Wang, Feng Cheng

**Affiliations:** 1 Department of Pharmaceutical Science, College of Pharmacy, University of South Florida, Tampa, Florida, United States of America; 2 School of Information, University of South Florida, Tampa, Florida, United States of America; 3 Department of Computer Science and Engineering, University of South Florida, Tampa, Florida, United States of America; 4 School of Biomedical Engineering, Tianjin Medical University, Tianjin, China; 5 Department of Epidemiology and Biostatistics, College of Public Health, University of South Florida, Tampa, United States of America; National Chiao Tung University College of Biological Science and Technology, TAIWAN

## Abstract

Identifying drug-drug interaction (DDI) is an important topic for the development of safe pharmaceutical drugs and for the optimization of multidrug regimens for complex diseases such as cancer and HIV. There have been about 150,000 publications on DDIs in PubMed, which is a great resource for DDI studies. In this paper, we introduced an automatic computational method for the systematic analysis of the mechanism of DDIs using MeSH (Medical Subject Headings) terms from PubMed literature. MeSH term is a controlled vocabulary thesaurus developed by the National Library of Medicine for indexing and annotating articles. Our method can effectively identify DDI-relevant MeSH terms such as drugs, proteins and phenomena with high accuracy. The connections among these MeSH terms were investigated by using co-occurrence heatmaps and social network analysis. Our approach can be used to visualize relationships of DDI terms, which has the potential to help users better understand DDIs. As the volume of PubMed records increases, our method for automatic analysis of DDIs from the PubMed database will become more accurate.

## Introduction

A drug-drug interaction (DDI) occurs when the pharmacologic effect of a given drug is altered by the action of another drug, leading to unpredictable clinical effects [1]. DDIs may make the drug less effective, delay drug absorption, or cause unexpected harmful side effects. Polypharmacy, the concurrent use of multiple medications, is an important factor for increasing the risk of DDIs. Centers for Disease Control (CDC) reported that the percentage of population taking three or more prescription drugs has increased from 11.8% in 1988–1994 to 20.8% in 2007–2010 in the United States. In addition, the percentage of people taking five or more drugs has increased from 4.0% to 10.1% during this same time period [2]. With each new drug added to an individual’s regime, the risk of DDIs may increase. Thus, DDI is becoming a serious clinical safety issue as the use of multiple medications becomes more common. DDIs account for more than 30% of all adverse drug events [3, 4]. In 2007, DDIs caused approximately 0.054% of emergency room visits, 0.57% of hospital admissions, and 0.12% of rehospitalizations in the United States [5]. DDI-related health care costs increase each year. For several drugs (e.g., cisapride, astemizol and terfenadine), the late identification of DDIs has led to restrictions, or even withdrawal of the drug from the market [6]. Therefore, detecting DDIs is an important topic to the pharmaceutical industry, drug regulatory agencies, healthcare professionals and patients [7].

DDI findings are frequently reported in clinical and scientific journals. PubMed developed by the National Library of Medicine (NLM), is the most widely used database of life sciences and biomedical literature. PubMed contains over 26 million entries from more than 5,600 journals, with 2,000–4,000 new references being added daily. PubMed is available for free on the Internet. A PubMed search for journal articles related to DDIs has produced about 150,000 results. This large amount of PubMed literature enables us to investigate DDIs comprehensively. However, it is difficult to read and summarize these references manually, even for specialists. Efficient and accurate retrieval of useful information is in increasing demand.

Bioinformatics approaches are widely used to investigate DDIs because of their ability to efficiently analyze large amounts of drug-related data including electronic health records [8], post-marketing safety surveillance reports [9–11], scientific literature [12–16], structural information on drug molecules [17–19] and drug-gene interactions [1, 20, 21]. In our previous paper [22], we developed a statistical method to identify compounds that might interact with the queried drug from substances information in MEDLINE records. Substances contain a lot of useful drug and compound information. In this study, we chose MeSH terms to systematically analyze DDIs and their mechanisms. MeSH terms, developed by the NLM, are used for indexing and annotating PubMed documents. MeSH terms are manually assigned to each document by biomedical subject specialists based on the context of the whole document. Thus, MeSH terms contain high-density information from the whole document which may not be inferred from the title or the abstract. In addition, MeSH terms are updated annually to include new vocabulary. In this paper, a random-sampling-based statistical algorithm was firstly applied to investigate the categories of drugs, proteins, and phenomena of MeSH terms. Co-occurrence heatmaps and networks were then plotted to explore the relationships among these terms. Three case studies on cyclosporine, rifampin and theophylline implied that our method was able to rank possible DDI-related terms with high accuracy. The co-occurrence heatmaps and social networks generated from these MeSH terms also illuminated possible associations among drugs, proteins, and phenomena, which can help people understand DDIs better. To our knowledge, there are no previous publications that have utilized these methods to study MeSH terms. Our computational approach will improve the ability of the research community to efficiently use increasingly large and complex PubMed data.

## Material and methods

### Identification of DDI-related MeSH terms

**Fig 1:**
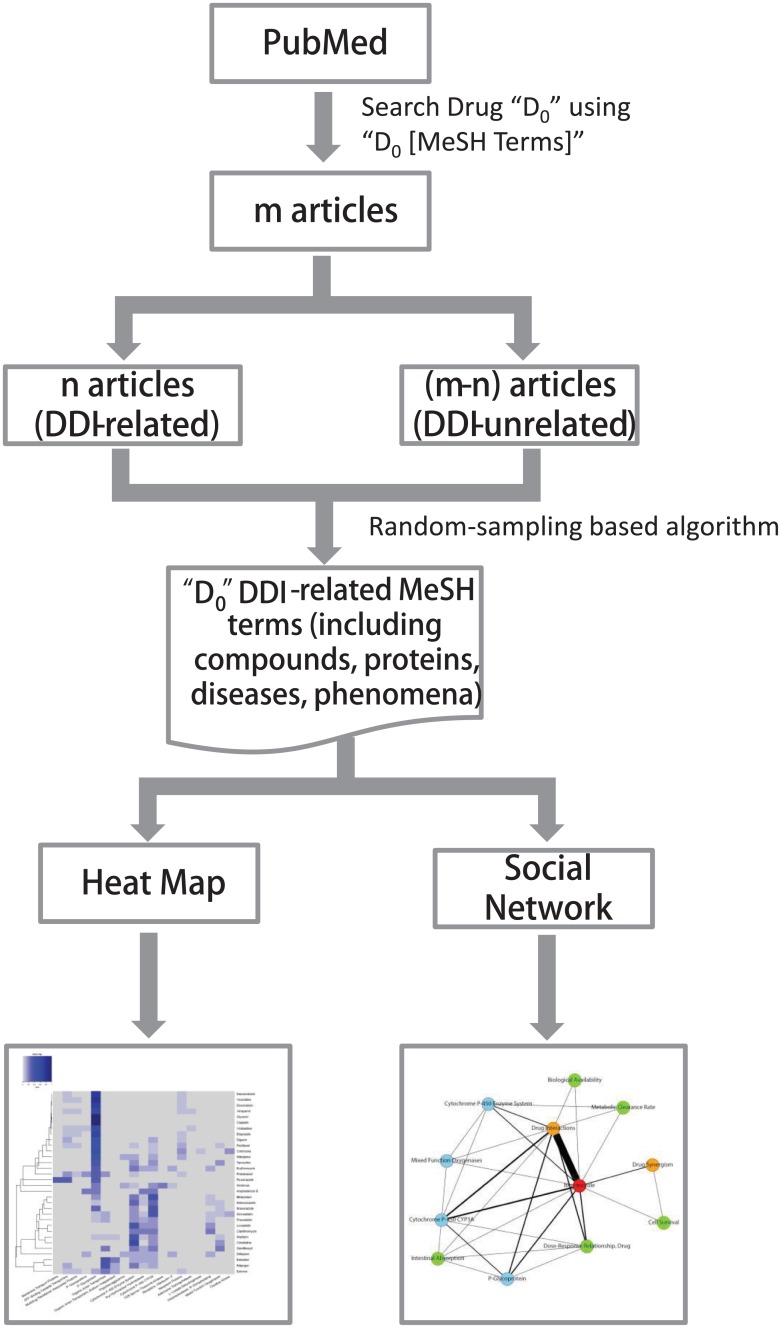
The workflow of the identification of significant MeSH terms.

The flowchart of the MeSH term analysis process is shown in Fig 1. In the first step, we searched PubMed using the query “Drug_0_ [MeSH Terms]” and downloaded the resulting articles. Here, “Drug_0_” was the name of a queried drug. Basic information including PubMed IDs (PMIDs), title, publication date, publication type, abstract, substances and MeSH terms fields of these articles was retrieved. The end of publication date was set to be 2015/12/31. In other words, only papers published before 2015/12/31 was included for data analysis. In the second step, the articles were divided into two groups, DDI-related literature (group A) and DDI-unrelated literature (group B). If a paper contained at least one of the drug interaction terms, it was selected as DDI-related literature. Drug interaction terms included “drug interactions”, “drug agonism”, “drug partial agonism”, “drug antagonism”, “drug inverse agonism”, “drug synergism”, “food-drug interactions”, and “herb-drug interactions”. The MeSH terms from DDI-related articles (group A) were extracted and chosen as candidate terms. 1,064 FDA-approved drugs [23] were found in the current version of the MeSH tree (2016 MeSH). These molecules were used to identify the drug terms. Terms in subgroups “D08” and “D12.776” in the MeSH tree were chosen as protein terms. MeSH terms in subgroups “G03”, “G04”, “G06” and “G07” were selected as phenomena terms. In the third step, a random-sampling based algorithm, reported in our previous paper [22], was applied to identify the MeSH terms whose frequency is significantly higher in DDI-related articles (group A) than frequency in DDI-unrelated articles (group B). In this step, the same number of articles in group B as articles in group A will be randomly selected. The number of candidate MeSH terms in these selected papers was counted. The process was repeated many times (for example, 1000 times) to establish the null distribution of the candidate term frequencies in the DDI-unrelated articles. The p-value for each candidate compound protein, and phenomena term was calculated based on the distribution using Z-statistics. Detailed information about the algorithm can be found in our previous study [22]. The terms whose frequency was more than 5 and p-value<0.1 were selected. The source python code for downloading papers from PubMed and random sampling can be accessed at the website: https://figshare.com/articles/step_1_download_Substance_py/4711516.

In the last step, to highlight the applicability of our random-sampling-based statistical algorithm, three medications, cyclosporine, rifampin (also known as rifampicin) and theophylline were chosen to validate our predictions of DDI-related drugs and proteins. Drug interactions are important for these compounds resulting in more than 1000 DDI-related articles in PubMed. Cyclosporine was introduced into clinical practice in the early 1980s as an effective immunosuppressant. Since that time, cyclosporine has been found to interact with many drugs which causes either elevated or subtherapeutic blood cyclosporine concentrations. Elevated cyclosporine levels have been linked to nephrotoxicity, neurotoxicity, and an increased risk of infection [24]. Rifampin is an important drug in the treatment of several types of bacterial infections. Rifampin is used extensively despite its broad effects on DDIs, creating serious problems. The clinical importance of such interactions includes auto induction leading to suboptimal or failed treatment [25]. Theophylline has been used to treat airway diseases for over 70 years. DDIs can alter blood theophylline concentrations. Elevated theophylline levels produce a wide range of adverse reactions including persistent vomiting, cardiac arrhythmias, and intractable seizures that can be lethal [26]. Drug interaction information about these drugs was collected from *LexiComp* (http://online.lexi.com), *Clinical Pharmacology (*http://clinicalpharmacology-ip.com), *Drug Interaction Checker* (http://www.drugs.com/drug_interactions.html), and *Micromedex* (http://micromedex.com/). These databases were chosen as the gold standard data for DDI validation. In addition, DDI-related proteins were manually validated from literatures. Receiver Operating Characteristic (ROC) curves, which show the sensitivity against one minus the specificity for all possible threshold values, were plotted to describe the ability of a model to correctly identify DDIs or proteins reported in the PubMed databases.

### Co-occurrence analysis of the relationships among significant DDI-related MeSH terms

Co-occurrence analysis was applied to explore the co-localization frequency of MeSH terms in the same paper. A term-article matrix was firstly built. The columns and rows of the matrix were terms and articles respectively. If term “A” exists in article “B”, the value in the matrix is set to be 1, otherwise, it is 0. The term-term adjacency matrix was then built from the term-article matrix, where the rows and columns represent terms, and every entry is the number of co-occurrences of the two terms. Co-occurrence heatmaps were plotted to visualize term-term adjacency matrix, where the numbers were substituted with colored cells. In the heatmap, Euclidean distance was used for hierarchical clustering. The heatmap can clearly show the co-occurrence of the DDI-related MeSH terms. A weighted network was also constructed with nodes representing MeSH terms and the edges representing interactions between two connected nodes. The co-occurrence heatmaps and term networks were plotted using gplot and igraph packages in R (https://www.r-project.org).

## Results

### Identification of DDI-related compounds and proteins from MeSH terms

**Fig 2:**
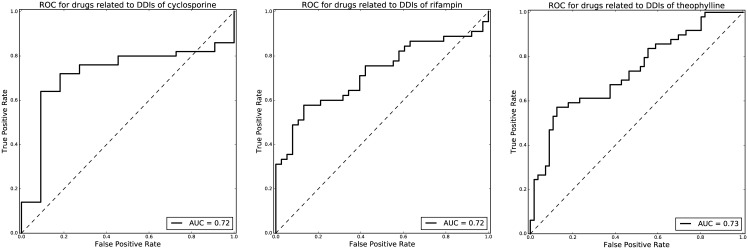
ROC curves for predicting DDI-related drug terms for cyclosporine, rifampin and theophylline.

As shown in Table 1, 1,996, 1,322 and 2,167 DDI-related articles containing drug interaction terms were found in PubMed for cyclosporine, rifampin and theophylline respectively. Fig 2 shows ROC curves for predicting DDI-related drug terms whose frequencies are greater than 5 times in DDI-related articles. The ROC curves from our model are closer to the upper left corner of the graph. The area under the curve (AUC) is 0.72 (cyclosporine), 0.72 (rifampin) and 0.73 (theophylline), which represents the accuracy of our model for the identification of DDIs. 46, 61 and 73 drug terms with p-value<0.1 and frequency greater than 5 times in DDI-related articles for cyclosporine, rifampin and theophylline were identified and listed in S1 Table. The comparison of ROC curves with the cutoff of term frequency ranging from 1 to 6 is shown in S1 Fig.

**Table 1:** Identification of significant DDI-related compounds and proteins from MeSH terms.

		Cyclosporine	Rifampin	Theophylline
Number of DDI-related articles	Including reviews	1996	1322	2167
	Without reviews	1804	1258	2038
Number of significant proteins	Including reviews	22	19	12
	Without reviews	22	18	12
Number of significant drugs	Including reviews	46	61	73
	Without reviews	41	58	70
AUC of ROC curves of drug terms	Including reviews	0.72	0.72	0.73
	without reviews	0.71	0.70	0.72
AUC of ROC curves of protein terms	Including reviews	0.9	0.86	0.79
	Without reviews	0.91	0.83	0.75

**Fig 3:**
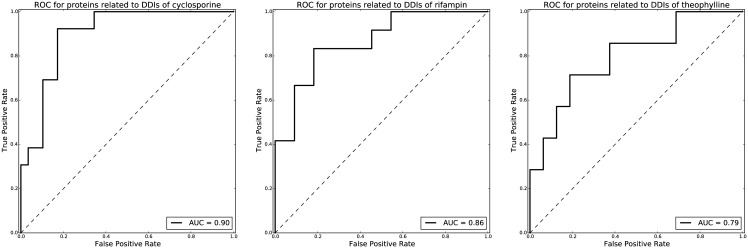
ROC curves for predicting DDI-related protein terms for cyclosporine, rifampin and theophylline.

The ROC curves for predicting DDI-related proteins terms whose frequencies are greater than 5 times in DDI-related articles were plotted in Fig 3. The AUC of the ROC curves is 0.9, 0.86, and 0.79 for cyclosporine, rifampin and theophylline which shows the model can correctly identify most of the DDI-related proteins from PubMed. As shown in Table 1 and S2 Table, 22, 19 and 12 statistically significant protein terms (p-value<0.1 and frequency greater than 5 times) were identified for cyclosporine, rifampin and theophylline using the same random-sampling-based approach. For cyclosporine, many DDI-related proteins are transporter proteins such as P-glycoprotein, ATP-binding cassette transporters, and organic anion transporters. Two cytochrome P-450 (CYP) isoenzyme CYP3A and CYP2E1 were also identified. For rifampin, several CYP isoenzymes (including CYP2C8, CYP3A, CYP2C9, CYP2D6, CYP2B6, and CYP1A2) and transporter proteins (including P-glycoprotein and organic anion transporters) were identified as proteins involved in DDIs. Additionally, glucuronosyltransferase was found. For theophylline, the majority of DDI-related proteins are CYP isoenzymes, including CYP1A2, CYP3A and CYP2E1. Phosphorylases and adrenergic receptors were also identified as proteins involved in DDIs.

### The co-occurrence of drugs and proteins

**Fig 4:**
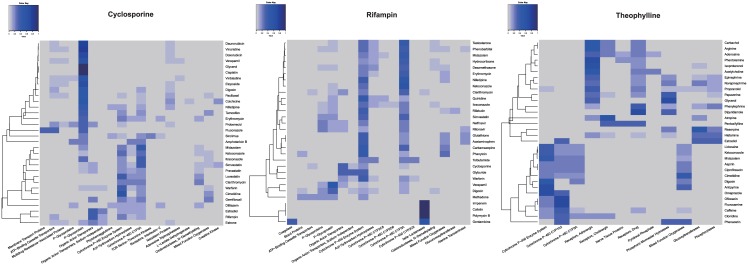
Protein-drug co-occurrence heatmaps for cyclosporine, rifampin and theophylline. The rows and columns represent drug and protein terms respectively, and each cell is the normalized count of co-occurrences of the two terms. The normalized count can be calculated as *normalized count = count/ total row count*.

Protein-drug co-occurrence heatmaps were plotted to display which proteins are involved in a specific DDI. As shown in Fig 4, for cyclosporine, the most frequent proteins are P-glycoprotein and CYP3A for most drug pairs. The most frequent DDI-related proteins are CYP3A and organic anion transporter for rifampin. Our results show that both cyclosporine and rifampin are mainly involved in pharmacokinetic DDIs, in which one drug affects the absorption, distribution, metabolism, or excretion (ADME) of another drug. The P-glycoprotein and organic anion transporter play an essential role in drug elimination and drug bioavailability [27]. For theophylline, the most frequently DDI-related proteins are CYP1A2, CYP3A, cholinergic receptors, and adrenergic receptors. Experiments showed that theophylline (or its metabolites) could stimulate cholinergic effect [28]. Beta-adrenergic blockers may also interfere the effects of theophylline if they are taken together [29].

### The co-occurrence of drugs and phenomena

**Fig 5:**
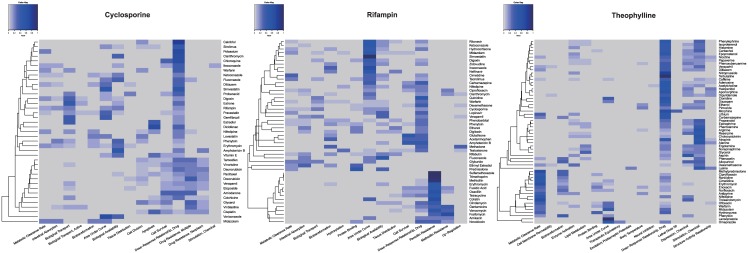
Phenomena-drug co-occurrence heatmaps for cyclosporine, rifampin and theophylline. The rows and columns represent drug and phenomena terms respectively, and each cell is the normalized count of co-occurrences of the two terms. The normalized count can be calculated as *normalized count = count/ total row count*.

The phenomena terms were also analyzed using the same approach as proteins. These phenomena terms contain important mechanism-related information including biological processes and clinical consequences. As shown in Fig 5, for cyclosporine, the most frequent phenomena include many pharmacokinetics-related terms, such as metabolic clearance rate, biological transport and biological availability. Similarly for rifampin, many pharmacokinetics-related terms including metabolic clearance rate, intestinal absorption, biotransformation, and biological availability were shown in the drug-phenomena heatmap. For theophylline, the majority of DDI-related phenomena were metabolic clearance rate, biotransformation, depression, and chemical stimulation.

### Network analysis of DDI-related terms

**Fig 6:**
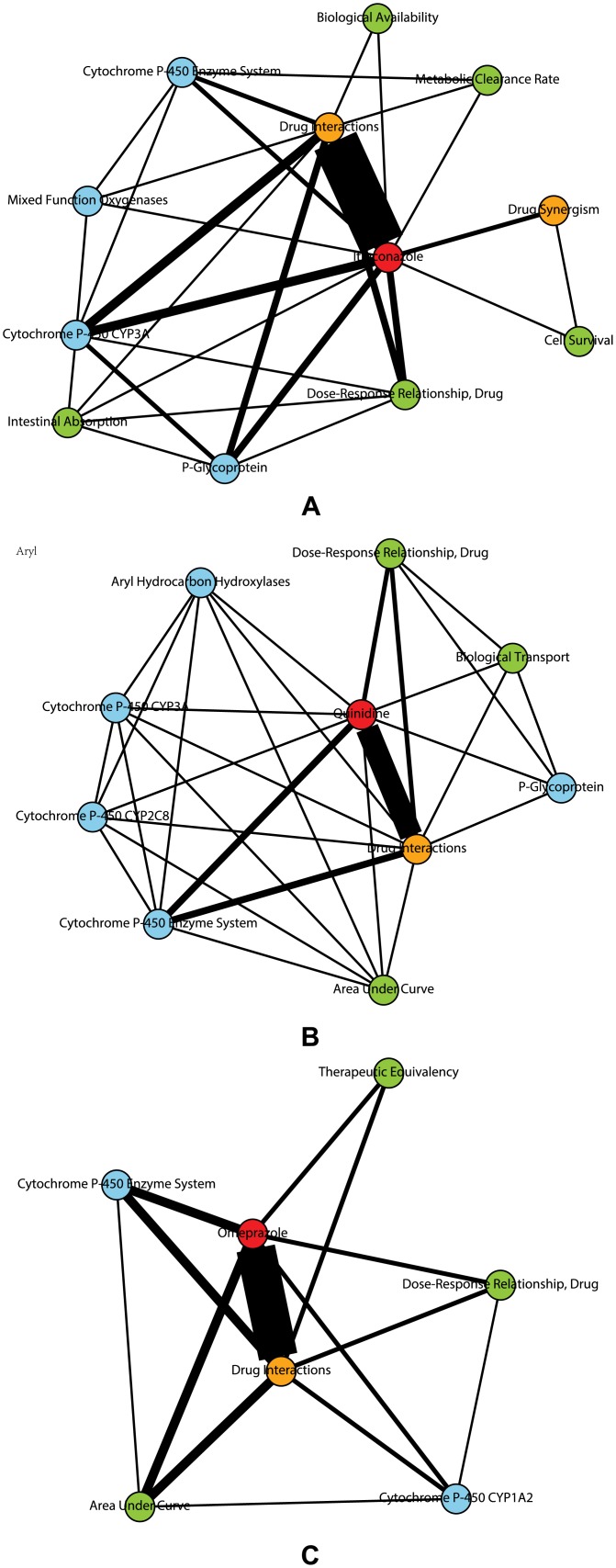
The social network of drug, proteins, phenomena and DDI types for three drug pairs, (A) cyclosporine-itraconazole, (B) rifampin-quindine, and (C) theophylline-omeprazole. Drugs, proteins, phenomena and DDI types are shown in red, blue, green and orange, respectively.

The connections among drugs, proteins and phenomena terms were modeled using social network analysis. Fig 6 shows the results for three drug pairs, cyclosporine-itraconazole, rifampin-quinidine, and theophylline-omeprazole. For cyclosporine-itraconazole (shown in Fig 6A), the proteins connecting to both drug interaction and itraconazole are CYP3A and P-Glycoprotein. The phenomena terms including biological availability, metabolic clearance rate, intestinal absorption, and drug dose-response relationship, are also connected to the terms drug interaction and itraconazole. These results are consistent with clinical observations that the metabolism of itraconazole can be affected when combined with cyclosporine. Cyclosporine can increase the level of itraconazole because itraconazole is eliminated from the body by P-glycoprotein and cyclosporine is the inhibitor of P-glycoprotein [30]. In addition, itraconazole can disturb the ADME of Cyclosporine. Cyclosporine is primarily metabolized by CYP3A4 [31], and itraconazole is an inhibitor of CYP3A4 [32]. As shown in Fig 6B, for rifampin-quinidine, MeSH terms including CYP3A, CYP2C8, P-Glycoprotein and dose-response relationship were connected to the terms drug interaction and quinidine. Clinical studies have found that rifampin strongly reduces plasma concentrations and the antiarrhythmic effects of quinidine by inhibiting CYP3A4 and P-glycoprotein [33, 34]. For theophylline-omeprazole (shown in Fig 6C), the terms connecting both drug interactions and omeprazole include CYP1A2, therapeutic equivalency, area under curve, and drug dose-response relationship. The results agree with the clinical findings that omeprazole may induce CYP1A2 and the induction of CYP1A2 can significantly reduce the plasma concentrations of theophylline [35].

### The effect of review articles on term identification

A review article in PubMed may contain duplicate content because it summarizes previously published research or clinical papers. To investigate the effect of review articles on the identification of DDI-related term, we performed the same analysis using the articles excluding review papers. Results were shown in Table 1. 192, 64, and 129 review papers were excluded for cyclosporine, rifampin and theophylline respectively. The number of identified MeSH terms and the AUCs of drugs and proteins ROC curves generally were similar or slightly decreased for cyclosporine, rifampin and theophylline.

## Discussion

PubMed was chosen as the literature resource for DDI studies in this project for three reasons. First, PubMed is the largest and the most widely used database of life sciences and biomedical literature. It contains more than 26 million scientific or clinical articles [22]. Among them, 150,000 articles are related to DDI. New references are added daily at an ever-increasing rate. The large amount of literature in PubMed enables us to comprehensively investigate DDIs and their molecular mechanisms. Second, PubMed is available for free on the Internet and original PubMed literature data can be easily downloaded. Third, PubMed records have a well-defined structure including title of the journal article, author information, journal information, publication type, language, abstract, MeSH terms, and substances. Information from the structured data can be efficiently analyzed by using computational methods. Among them, MeSH terms provide high-density information from the whole article.

Our method automatically explored the possible DDI-related MeSH terms and the relationships among them from the literature in the PubMed database. Our approach has the following advantages. First, current literature-based approaches are generally performed to discover DDIs from scientific literature by text mining [12–16]. To the best of our knowledge, our method is the first automatic approach that can systematically analyze the DDIs directly from MeSH terms. As the volume of records in the PubMed database grows, manual curation techniques become increasingly less desirable and our approach will become more important. Second, the random sampling step in our algorithm can effectively filter unrelated DDI terms. As shown in ROC curves, many DDIs and DDI-related proteins identified from our method have been validated. Third, we performed a systematic analysis of MeSH terms, including drugs, proteins, and phenomena, of PubMed records to explore the mechanism of DDIs. These fields contain important information related to DDIs. Fourth, our approach can show the relationship among these terms by using co-occurrence heatmaps and social networks, which provides a novel way to visualize and explore possible connections among terms without priori assumptions.

Nonetheless, there are some limitations to the present study. First, some important information in PubMed articles may not be collected because MeSH terms are manually summarized [36]. Additionally, the identified MeSH terms are not from the exact content of the text and the associations identified among MeSH terms are indirect. Therefore, the findings can only be hypotheses but not proofs. In the future, we plan to apply Natural Language Processing (NLP) tools to automatically index more terms and to identify direct associations from the abstract or the full text of PubMed literature. Second, there are only 1,064 FDA approved drugs in the MeSH tree. Some drugs are not available in MeSH terms. Therefore, we missed DDIs involving the drugs not included in the MeSH tree. This limitation of the study can be overcome if more MeSH terms are included in the future. Third, the number of papers for some drugs/compounds is few and some low-frequency but important DDIs are excluded. However, PubMed records increase rapidly and more DDI information can be identified by our method in the future.

## Conclusion

In this paper, we have described a method for analyzing the action mechanism of DDIs based on automated extraction of relevant MeSH terms from PubMed. The method can identify DDI-related terms including compounds, proteins and phenomena, and help users to visualize relationships of these terms using co-occurrence heatmaps and social networks. The success of the project will open doors to the future use of similar techniques in literature analysis.

## Supporting information

S1 Fig Comparison of ROC curves for predicting DDI-related drug terms for cyclosporine, rifampin and theophylline with the cutoff of term frequency ranging from 1 to 6. (PDF)

S1 Table Significant DDI-related drug terms for cyclosporine, rifampin and theophylline. The gold standard in the manuscript is known DDI pairs from four databases including LexiComp, Clinical Pharmacology, Drug Interaction Checker, and Micromedex. “Actual result” indicates whether the identified drug term is true (1) or false (0).
(PDF)

S2 Table Significant DDI-related proteins for cyclosporine, rifampin and theophylline. DDI-related proteins were manually validated from literatures. “Actual result” indicates whether the identified protein term is true (1) or false (0).
(PDF)

S3 Table PRISMA 2009 checklist. (DOC)
