# Supplementary figures and images for: Characterization of the mechanism of drug-drug interactions from PubMed using MeSH terms
Source: PLoS One. 2017 Apr 19;12(4):e0173548. doi: 10.1371/journal.pone.0173548 (PMC5396881; doi:10.1371/journal.pone.0173548)

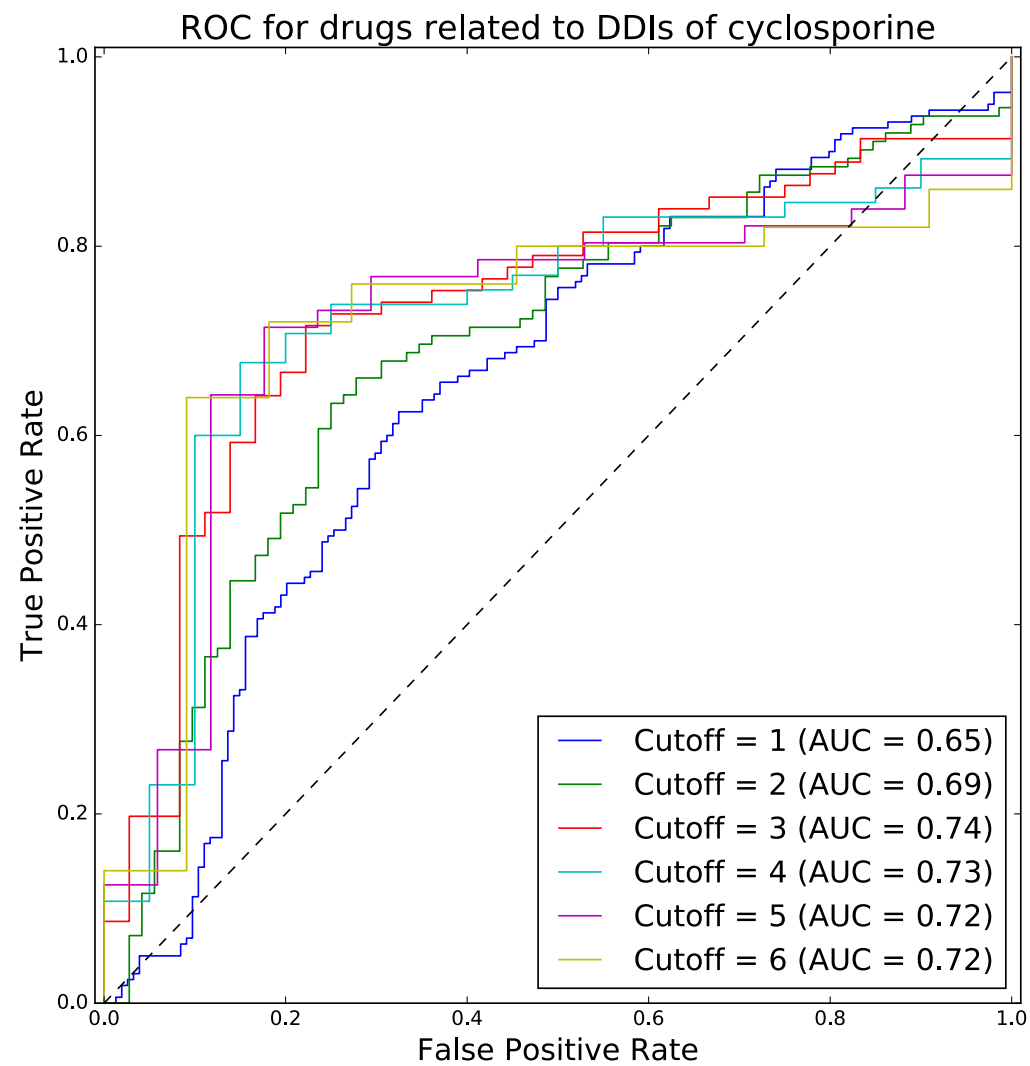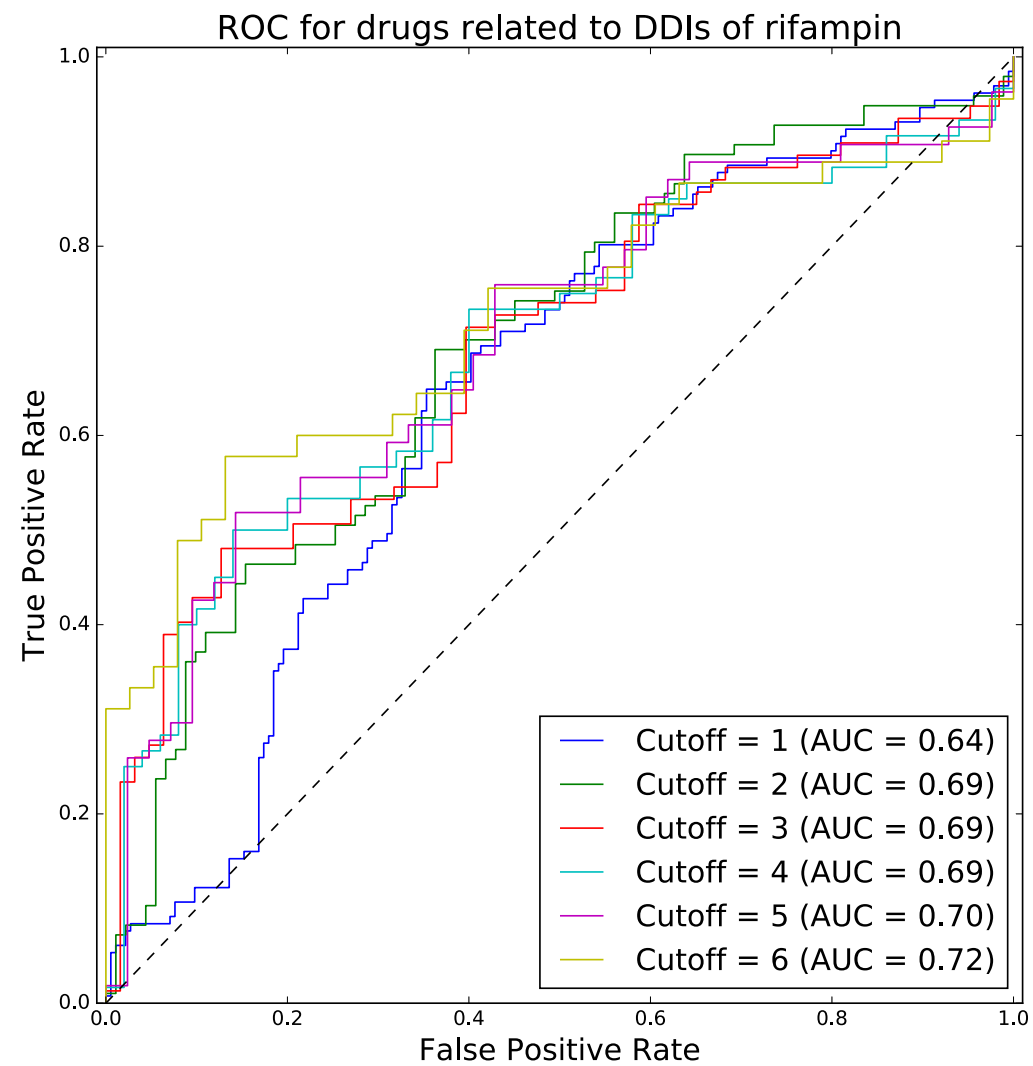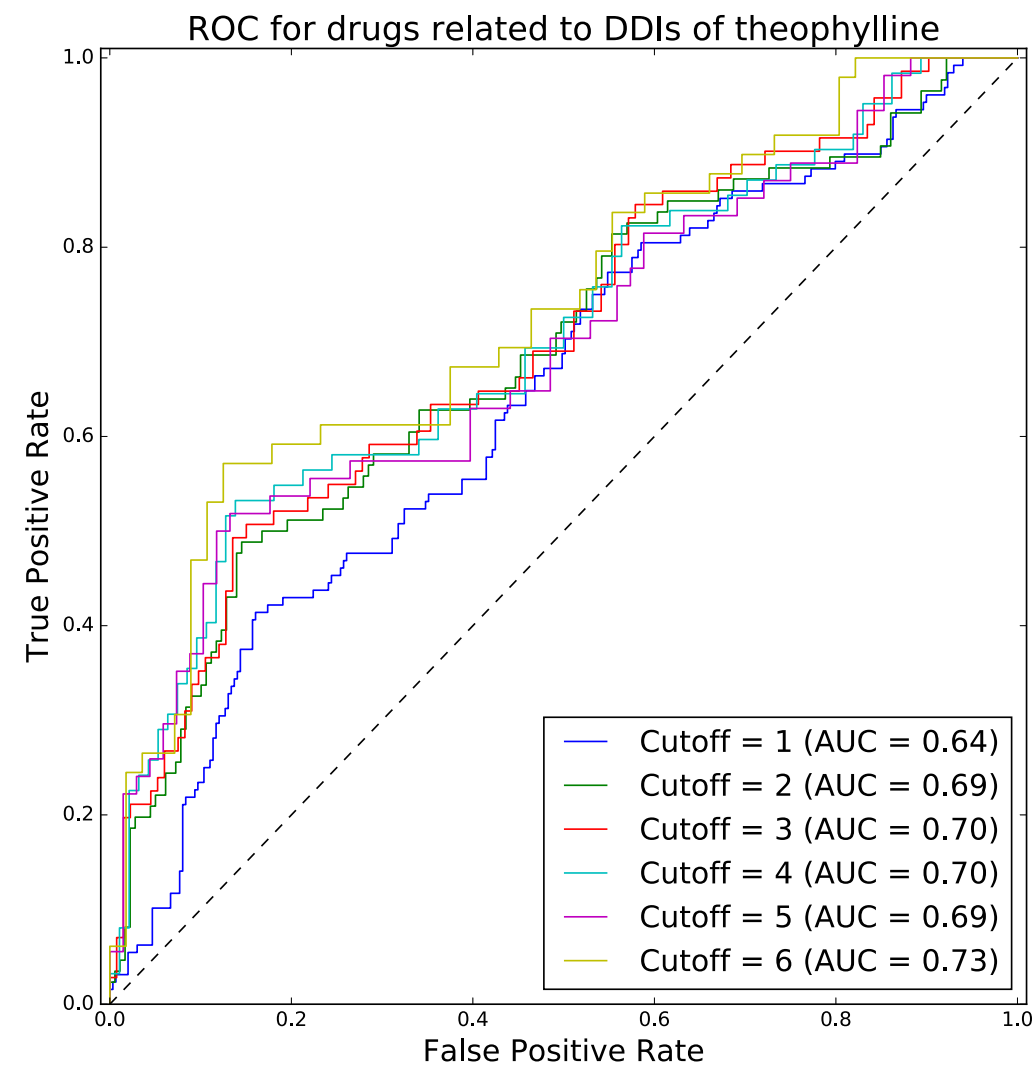

Supplement: S1 Fig — (PDF) [file pone.0173548.s001.pdf]
