# Supplementary material for: Characterization of the mechanism of drug-drug interactions from PubMed using MeSH terms
Source: PLoS One. 2017 Apr 19;12(4):e0173548. doi: 10.1371/journal.pone.0173548 (PMC5396881; doi:10.1371/journal.pone.0173548)
Supplement: S1 Table — The gold standard in the manuscript is known DDI pairs from four databases including LexiComp, Clinical Pharmacology, Drug Interaction Checker, and Micromedex. “Actual result” indicates whether the identified drug term is true (1) or false (0). (PDF) [file pone.0173548.s002.pdf]

S1 Table. Significant DDI-related drug terms for cyclosporine, rifampin and theophylline. The gold standard in the manuscript is known DDI pairs from four databases including LexiComp, Clinical Pharmacology, Drug Interaction Checker, and Micromedex. "Actual result" indicates whether the identified drug term is true (1) or false (0).

Cyclosporine

Drug Name	p value	Micromedex	Lexicomp	Clinical Pharmacology	Drug Checker	Actual result
Voriconazole	0	Major	Null	Level 2(Major)	Major	1
Rifampin	0	Major	Null	Level 2(Major)	Major	1
Fluconazole	0	Major	Null	Level 2(Major)	Moderate	1
Itraconazole	0	Major	Null	Level 2(Major)	Major	1
Ketoconazole	0	Moderate	Consider therapy modification	Level 2(Major)	Major	1
Simvastatin	0	Contraindicated	Null	Level 1(Severe)	Major	1
Warfarin	0	Moderate	Null	Level 3(Moderate)	Moderate	1
Estrone	0	Null	Null	Null	Null	0
Gemfibrozil	0	Null	Null	Null	Moderate	1
Digoxin	0	Moderate	Null	Level 2(Major)	Moderate	1
Clarithromycin	0	Moderate	Null	Level 2(Major)	Major	1
Probucol	0	Moderate	Null	Level 3(Moderate)	Moderate	1
Pravastatin	0	Major	Null	Null	Moderate	1
Erythromycin	0	Moderate	Monitor therapy	Level 2(Major)	Major	1
Cimetidine	0	Moderate	Null	Level 3(Moderate)	Minor	1
Doxorubicin	0	Major	Consider therapy modification	Level 2(Major)	Moderate	1
Verapamil	0	Moderate	Null	Level 3(Moderate)	Moderate	1
Midazolam	0	Moderate	Null	Null	Null	1
Diltiazem	0	Moderate	Null	Level 3(Moderate)	Moderate	1
Felodipine	0	Major	Null	Level 2(Major)	Moderate	1
Nifedipine	1.22E-15	Null	Null	Level 3(Moderate)	Moderate	1
Chloroquine	2.57E-13	Moderate	Null	Null	Moderate	1
Amphotericin B	7.02E-13	Moderate	Monitor therapy	Level 3(Moderate)	Moderate	1
Sirolimus	1.36E-12	Moderate	Null	Level 2(Major)	Moderate	1
Vincristine	1.62E-11	Major	Avoid combination	Null	Moderate	1
Probenecid	5.64E-11	Null	Null	Null	Major	1
Paclitaxel	1.61E-10	Null	Monitor therapy	Level 2(Major)	Moderate	1
Diclofenac	1.94E-10	Major	Consider therapy modification	Level 2(Major)	Moderate	1
Vinblastine	6.95E-10	Null	Null	Null	Minor	1
Lovastatin	1.11E-08	Major	Null	Null	Major	1
Colchicine	4.55E-08	Contraindicated	Null	Level 2(Major)	Major	1

Amiodarone	6.59E-08	Moderate	Null	Level 3(Moderate)	Moderate	1
Daunorubicin	2.75E-07	Null	Monitor therapy	Level 2(Major)	Moderate	1
Tamoxifen	5.12E-07	Null	Null	Null	Null	0
Estradiol	2.06E-06	Null	Monitor therapy	Null	Moderate	1
Losartan	3.27E-05	Null	Null	Null	Moderate	1
Indomethacin	0.000119002	Major	Null	Null	Moderate	1
Vitamin E	0.000243027	Null	Monitor therapy	Null	Minor	1
Calcitriol	0.000560794	Null	Null	Null	Null	0
Etoposide	0.000718607	Major	Null	Null	Moderate	1
Cisplatin	0.002769689	Null	Null	Level 3(Moderate)	Null	1
Glycerol	0.004404986	Null	Null	Null	Null	0
Acetylcholine	0.008687497	Null	Null	Null	Null	0
Thalidomide	0.0144237	Moderate	Null	Null	Null	1
Phenytoin	0.063315229	Moderate	Null	Level 3(Moderate)	Moderate	1
Potassium	0.077725096	Null	Null	Null	Null	0

Rifampin

Drug name	p value	Micromedex	Lexicomp	Clinical Pharmacology	Drug Checker	Actual result
Nelfinavir	0	Contraindicated	Null	Null	Major	1
Ketoconazole	0	Major	Consider therapy modification	Level 2(Major)	Moderate	1
Simvastatin	0	Moderate	Null	Null	Major	1
Warfarin	0	Moderate	Null	Level 2(Major)	Major	1
Digoxin	0	Moderate	Null	Level 3(Moderate)	Moderate	1
Tacrolimus	0	Major	Consider therapy modification	Level 2(Major)	Major	1
Cyclosporine	0	Major	Consider therapy modification	Level 2(Major)	Major	1
Methadone	0	Moderate	Null	Level 2(Major)	Moderate	1
Phenytoin	0	Major	Null	Level 2(Major)	Moderate	1
Digitoxin	0	Moderate	Null	Null	Moderate	1
Itraconazole	0	Major	Null	Level 2(Major)	Moderate	1
Ritonavir	0	Major	Null	Null	Major	1
Verapamil	0	Moderate	Consider therapy modification	Null	Moderate	1
Glyburide	0	Moderate	Null	Null	Moderate	1
Amphotericin B	0	Null	Null	Null	Null	0
Midazolam	0	Moderate	Null	Level 2(Major)	Minor	1
Colistin	0	Null	Null	Null	Null	0
Fluconazole	0	Moderate	Null	Level 2(Major)	Moderate	1
Cimetidine	0	Null	Null	Null	Null	0
Ethanol	0	Null	Null	Level 2(Major)	Null	1
Lopinavir	0	Contraindicated	Null	Null	Null	1

Nifedipine	0	Major	Avoid combination	Level 1(Severe)	Moderate	1
Carbamazepine	0	Moderate	Null	Level 2(Major)	Moderate	1
Nevirapine	0	Major	Consider therapy modification	Level 1(Severe)	Major	1
Quinidine	0	Moderate	Null	Level 2(Major)	Moderate	1
Imipenem	0	Null	Null	Null	Null	0
Theophylline	0	Moderate	Null	Null	Moderate	1
Fosfomycin	0	Null	Null	Null	Null	0
Zidovudine	0	Moderate	Null	Null	Moderate	1
Tolbutamide	0	Moderate	Null	Null	Moderate	1
Phenobarbital	9.99E-16	Null	Null	Level 2(Major)	Moderate	1
Rifabutin	1.20E-12	Null	Null	Null	Null	0
Ceftazidime	1.28E-12	Null	Null	Null	Null	0
Pefloxacin	5.44E-12	Null	Null	Null	Null	0
Prednisolone	3.63E-11	Moderate	Monitor therapy	Null	Moderate	1
Polymyxin B	9.41E-11	Null	Null	Null	Null	0
Gentamicins	3.75E-10	Null	Null	Null	Null	0
Phenylbutazone	6.29E-10	Null	Null	Null	Null	0
Novobiocin	1.45E-09	Null	Null	Null	Null	0
Ethinyl Estradiol	2.70E-09	Moderate	Null	Null	Major	1
Vancomycin	4.63E-09	Null	Null	Null	Null	0
Hydrocortisone	1.38E-07	Moderate	Monitor therapy	Null	Moderate	1
Oxacillin	1.41E-06	Null	Null	Null	Null	0
Flucytosine	4.91E-06	Null	Null	Null	Null	0
Trimethoprim	1.15E-05	Null	Null	Level 3(Moderate)	Moderate	1
Acetaminophen	7.83E-05	Null	Null	Level 3(Moderate)	Minor	1
Clarithromycin	0.000132095	Moderate	Null	Level 2(Major)	Moderate	1
Antipyrine	0.000149341	Null	Null	Null	Null	0
Ciprofloxacin	0.000181653	Null	Monitor therapy	Null	Null	1
Testosterone	0.000660474	Null	Null	Null	Moderate	1
Methicillin	0.001190581	Null	Null	Null	Null	0
Clindamycin	0.001536213	Null	Null	Null	Null	0
Fusidic Acid	0.00160905	Null	Null	Null	Null	0
Bacitracin	0.001947541	Null	Null	Null	Null	0
Amikacin	0.002012159	Null	Null	Null	Null	0
Erythromycin	0.002900355	Null	Consider therapy modification	Level 2(Major)	Moderate	1
Tobramycin	0.012559574	Null	Null	Null	Null	0
Sulfamethoxazole	0.035978179	Null	Null	Level 3(Moderate)	Moderate	1
Dexamethasone	0.050438031	Moderate	Consider therapy modification	Null	Moderate	1
Glutathione	0.056053813	Null	Null	Null	Null	0
Tetracycline	0.090097233	Null	Null	Null	Moderate	1

Theophylline

Drug Name	p value	Micromedex	Lexicomp	Clinical Pharmacology	Drug Checker	Actual result
Ciprofloxacin	0	Major	Consider therapy modification	Level 2(Major)	Major	1
Cimetidine	0	Major	Null	Level 2(Major)	Moderate	1
Erythromycin	0	Major	Consider therapy modification	Level 2(Major)	Moderate	1
Warfarin	0	Null	Null	Null	Null	0
Fluvoxamine	0	Major	Null	Level 2(Major)	Major	1
Clarithromycin	0	Minor	Null	Level 2(Major)	Moderate	1
Ofloxacin	0	Minor	Consider therapy modification	Null	Moderate	1
Ranitidine	0	Minor	Null	Level 3(Moderate)	Moderate	1
Enoxacin	0	Major	Null	Null	Major	1
Norfloxacin	0	Moderate	Null	Null	Moderate	1
Omeprazole	0	Null	Null	Null	Moderate	1
Mexiletine	0	Major	Null	Level 3(Moderate)	Moderate	1
Lansoprazole	0	Minor	Null	Level 4(Minor)	Moderate	1
Terfenadine	0	Null	Null	Null	Null	0
Midazolam	0	Moderate	Null	Null	Minor	1
Pimozide	0	Null	Null	Null	Null	0
Apomorphine	1.11E-16	Null	Null	Null	Null	0
Troleandomycin	1.11E-16	Major	Null	Level 2(Major)	Moderate	1
Isoniazid	3.33E-16	Minor	Null	Null	Moderate	1
Norepinephrine	2.22E-15	Null	Null	Null	Null	0
Rifampin	8.25E-14	Moderate	Null	Level 2(Major)	Moderate	1
Carbamazepine	2.34E-13	Moderate	Null	Level 2(Major)	Moderate	1
Isoproterenol	3.22E-12	Moderate	Null	Null	Moderate	1
Epinephrine	1.20E-10	Null	Monitor therapy	Null	Null	1
Lithium	5.85E-10	Moderate	Null	Level 2(Major)	Moderate	1
Propranolol	8.61E-10	Moderate	Null	Null	Major	1
Nifedipine	8.96E-10	Minor	Null	Null	Minor	1
Phenytoin	1.05E-09	Major	Null	Level 3(Moderate)	Moderate	1
Histamine	4.19E-09	Null	Null	Null	Null	0
Ketoconazole	6.71E-09	Null	Consider therapy modification	Level 4(Minor)	Minor	1
Allopurinol	8.57E-09	Moderate	Null	Level 4(Minor)	Minor	1
Ergotamine	1.23E-08	Null	Null	Null	Moderate	1
Reserpine	7.21E-08	Null	Null	Null	Null	0
Diltiazem	1.57E-07	Moderate	Null	Level 3(Moderate)	Moderate	1
Terbutaline	1.03E-05	Minor	Null	Null	Moderate	1
Hydroxyurea	1.21E-05	Null	Null	Null	Null	0
Haloperidol	1.61E-05	Null	Null	Null	Null	0

Glycerol	2.41E-05	Null	Null	Null	Null	0
Pentoxifylline	4.02E-05	Moderate	Null	Null	Moderate	1
Morphine	0.000101897	Null	Null	Null	Null	0
Nicotine	0.000116199	Null	Null	Null	Null	0
Antipyrine	0.000123078	Null	Null	Null	Null	0
Diazepam	0.000142808	Moderate	Null	Null	Minor	1
Clonidine	0.000218889	Null	Null	Null	Null	0
Phenoxybenzamine	0.000222873	Null	Null	Null	Null	0
Aspirin	0.00024491	Null	Null	Null	Null	0
Lysine	0.000362401	Null	Null	Null	Null	0
Phentolamine	0.000503951	Null	Null	Null	Null	0
Cholecystokinin	0.000571972	Null	Null	Null	Null	0
Tetracycline	0.000623148	Null	Null	Null	Null	0
Phenacetin	0.000806017	Null	Null	Null	Null	0
Dipyridamole	0.000918772	Null	Null	Null	Moderate	1
Phenylephrine	0.001167878	Null	Monitor therapy	Null	Moderate	1
Halothane	0.001232781	Major	Null	Null	Major	1
Ethanol	0.002312524	Null	Null	Null	Null	0
Epoprostenol	0.003745631	Null	Null	Null	Null	0
Digoxin	0.003956863	Null	Null	Null	Null	0
Verapamil	0.004940374	Moderate	Null	Level 3(Moderate)	Moderate	1
Alanine	0.005125899	Null	Null	Null	Null	0
Acetylcholine	0.005204899	Null	Null	Null	Null	0
Lidocaine	0.006622202	Null	Null	Null	Moderate	1
Caffeine	0.007648404	Null	Null	Level 2(Major)	Moderate	1
Arginine	0.010821092	Null	Null	Null	Null	0
Papaverine	0.015567569	Null	Null	Null	Null	0
Nitroprusside	0.01814649	Null	Null	Null	Null	0
Prednisolone	0.023412626	Null	Null	Null	Moderate	1
Fenoterol	0.028820318	Null	Null	Null	Null	0
Methylprednisolone	0.035004248	Null	Null	Null	Moderate	1
Dexamethasone	0.042006899	Null	Monitor therapy	Null	Moderate	1
Atropine	0.042072221	Null	Null	Null	Null	0
Adenosine	0.075564767	Major	Null	Level 2(Major)	Moderate	1
Estradiol	0.077674371	Null	Monitor therapy	Null	Moderate	1
Carbachol	0.098154922	Null	Null	Null	Null	0
