# Supplementary material for: Characterization of the mechanism of drug-drug interactions from PubMed using MeSH terms
Source: PLoS One. 2017 Apr 19;12(4):e0173548. doi: 10.1371/journal.pone.0173548 (PMC5396881; doi:10.1371/journal.pone.0173548)
Supplement: S2 Table — DDI-related proteins were manually validated from literatures. “Actual result” indicates whether the identified protein term is true (1) or false (0). (PDF) [file pone.0173548.s003.pdf]

S2 Table. Significant DDI-related proteins for cyclosporine, rifampin and theophylline. DDI-related proteins were manually validated from literatures. "Actual result" indicates whether the identified protein term is true (1) or false (0).

Cyclosporine

Protein name	p value	Actual result
Organic Anion Transporters	0	1
Cytochrome P-450 CYP3A	0	1
P-Glycoprotein	0	1
Cytochrome P-450 Enzyme System	0	1
Mixed Function Oxygenases	0	0
Organic Anion Transporters, Sodium-Independent	0	1
Aryl Hydrocarbon Hydroxylases	0	0
Oxidoreductases, N-Demethylating	7.18E-14	0
ATP-Binding Cassette Transporters	1.68E-09	1
Multidrug Resistance-Associated Proteins	1.88E-08	1
Membrane Transport Proteins	6.75E-08	1
Creatine Kinase	3.33E-07	1
Immunotoxins	8.54E-07	0
Adenosine Triphosphatases	7.17E-06	0
Phytohemagglutinins	8.36E-05	1
Neoplasm Proteins	0.000242148	1
P-Glycoproteins	0.000268303	1
Potassium Channels	0.002236825	0
L-Lactate Dehydrogenase	0.00950413	0
TOR Serine-Threonine Kinases	0.03366479	0
Cholesterol, LDL	0.057253658	0
Receptors, Interleukin-2	0.060776758	0

Rifampin

Protein name	p value	Actual result
Cytochrome P-450 CYP3A	0	1
Cytochrome P-450 CYP2C8	0	1
Organic Anion Transporters	0	1
Cytochrome P-450 Enzyme System	0	1
P-Glycoprotein	0	1
Cytochrome P-450 CYP2D6	0	0
Cytochrome P-450 CYP2C9	2.78E-15	1
beta-Lactamases	1.99E-14	1
P-Glycoproteins	4.45E-14	1
Aryl Hydrocarbon Hydroxylases	5.53E-14	0

Organic Anion Transporters, Sodium-Independent	1.45E-13	1
Cytochrome P-450 CYP2B6	2.77E-11	1
Oxidoreductases, N-Demethylating	2.83E-05	0
Coagulase	0.000269775	0
Mixed Function Oxygenases	0.000325094	0
Glucuronosyltransferase	0.000384339	1
Blood Proteins	0.010634945	0
ATP-Binding Cassette Transporters	0.014110964	1
Alanine Transaminase	0.069149019	0

Theophylline

Protein name	p value	Actual result
Cytochrome P-450 CYP1A2	5.77E-15	1
Phosphorylases	1.40E-12	1
Glucosyltransferases	2.13E-10	0
Cytochrome P-450 Enzyme System	1.34E-07	1
Receptors, Cholinergic	2.61E-07	0
Cytochrome P-450 CYP3A	1.64E-05	1
Phosphoric Monoester Hydrolases	0.000260075	0
Mixed Function Oxygenases	0.000309117	1
Receptors, Drug	0.000982719	0
Pyridoxal Phosphate	0.009505123	0
Nerve Tissue Proteins	0.027571582	0
Receptors, Adrenergic	0.038638602	1
